# Bilateral and Simultaneous Rupture of the Triceps Tendon in a Patient without Predisposing Factors

**DOI:** 10.1155/2012/920685

**Published:** 2012-10-14

**Authors:** Bobby Desai, John Slish, Brandon Allen

**Affiliations:** Department of Emergency Medicine, University of Florida College of Medicine, 1329 SW 16th Street, P.O. Box 100186, Gainesville, FL 32610-0186, USA

## Abstract

Tendon rupture is typically associated with predisposing features including renal failure, hyperparathyroidism, and connective tissue elastosis. We present a case in which none of these risk factors is present and in a completely healthy patient. To our knowledge, this has never been reported in the literature.

## 1. Case Presentation

A 43-year-old male presented to our emergency department with complaints of elbow injury. The patient was wake boarding in the ocean and subsequently fell into the water. In order to break his fall, he threw his arms out in front of him. After the event, he noted pain in both elbows and noted decreased strength in both elbow extensors. He noted the right elbow to be worse than the left. The patient also noted a “knot” near the elbow on the right that was not present prior to the event. Furthermore, the patient denied loss of consciousness, no neck pain, and any other complaints. On social history, the patient is a healthy police officer, who does not take any medications and does not smoke or does illicit drugs.

On physical exam, the patient's vital signs were unremarkable with a normal temperature, pulse, respiratory rate, and blood pressure. The patient's physical exam was completely normal except for his extremity exam. The patient had normal radial pulses and normal capillary refill. He also had a normal sensory exam to pinprick and light touch. Motor exam revealed normal 5/5 flexor strength in bilateral upper extremities. Inspection revealed preserved lateral triceps head integrity, tension during extension on both sides, and swelling near the elbow which were thought to be tendon attachments. On formal extensor motor exam, the patient was barely able to overcome gravity on both the right and left sides. He was noted to have minimal engagement of the medial and long heads of both triceps. The examination was noted to be difficult due to the bilateral nature of the injury and the inability to compare the affected sides.

**Figure 1:**
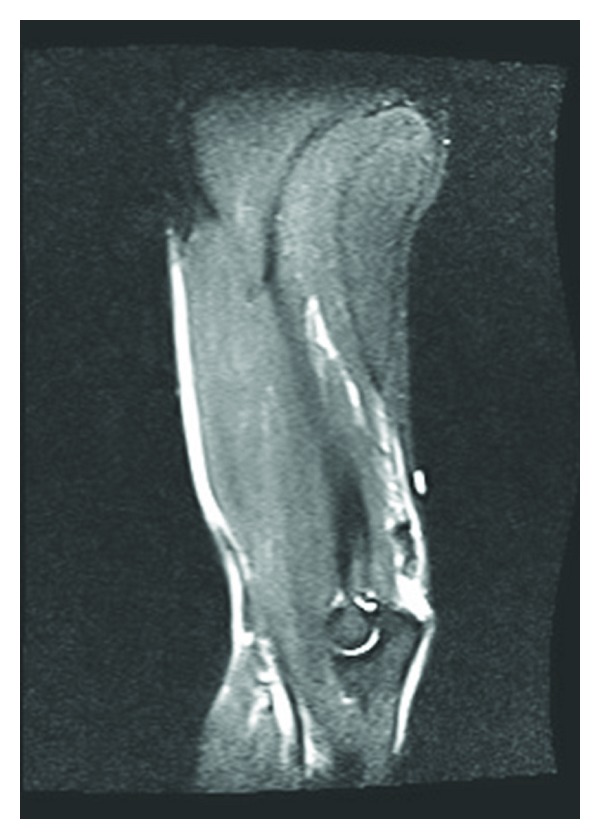
Left elbow, avulsion fracture through an enthesophyte at the distal triceps insertion, with 2.6 cm of proximal retraction of the triceps tendon. The underlying triceps ligament is thickened and of abnormal signal consistent with triceps tendinopathy. Ulnar collateral ligament stripping injury off the sublime tubercle. The remaining ligaments of the elbow are intact.

**Figure 2:**
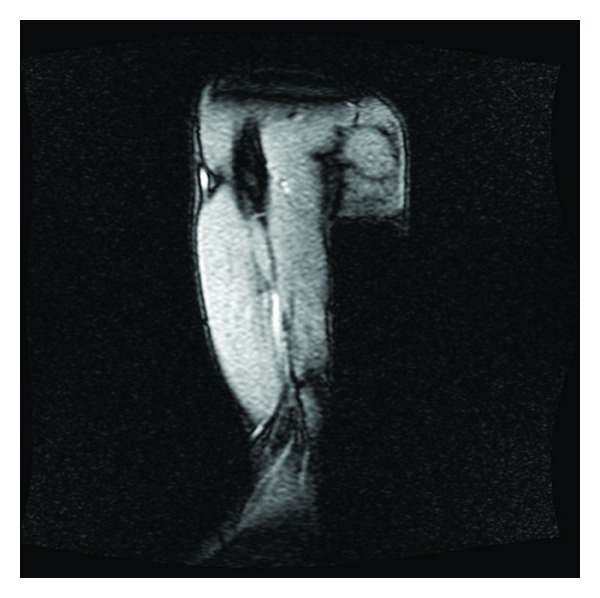
Same elbow, different view.

**Figure 3:**
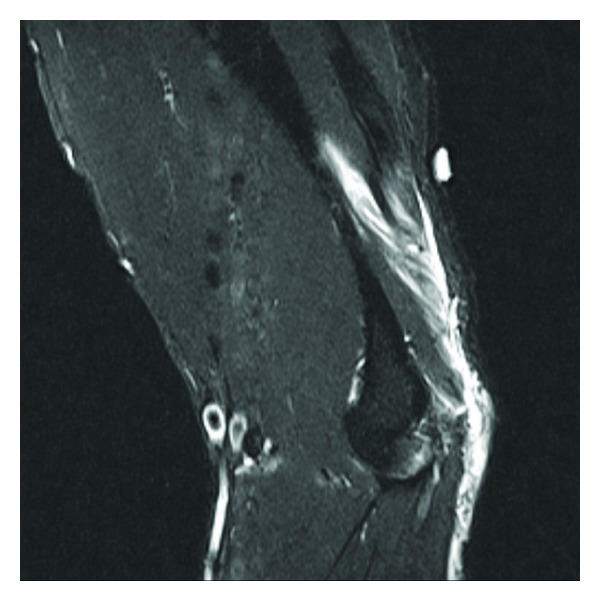
Right elbow, avulsion fracture through an enthesophyte at the triceps tendon insertion on the olecranon process of the right proximal ulna, with 9 mm of proximal retraction. There is underlying chronic tendinosis of the triceps tendon. No additional fractures are identified.

**Figure 4:**
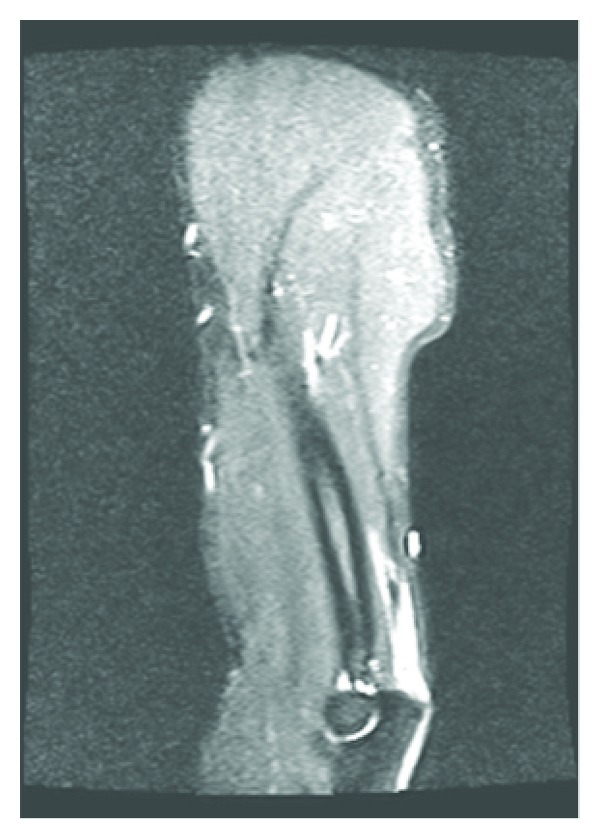
Same elbow, different view.

**Figure 5:**
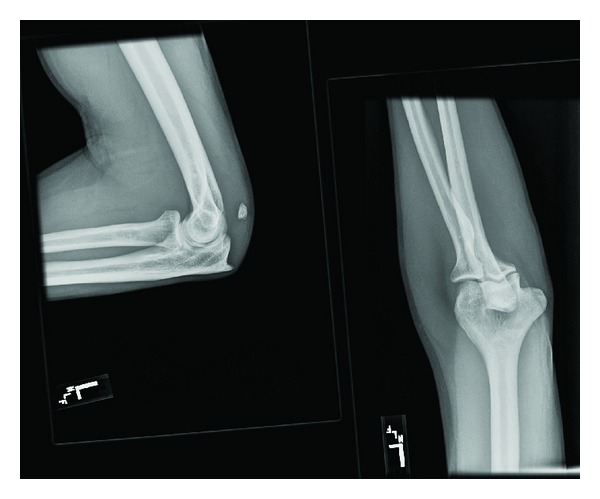
Plain radiograph, left elbow with avulsion fracture.

**Figure 6:**
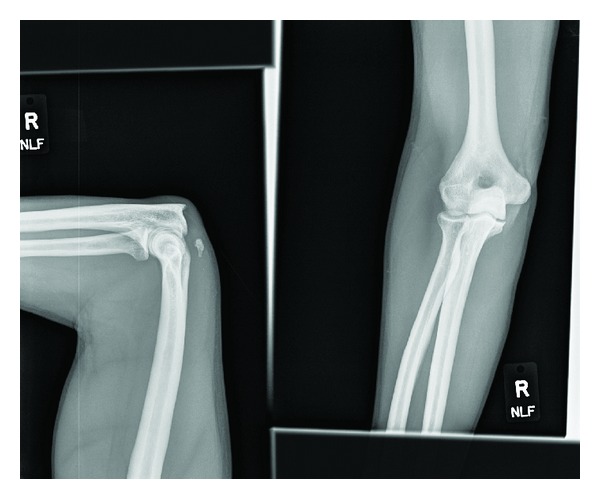
Plain radiograph, right elbow with avulsion fracture.

Bilateral plain radiographs of the elbow were ordered. The right elbow radiograph showed an avulsion fracture of the right olecranon with no dislocation. The left elbow radiograph was the same, showing an avulsion fracture of the left olecranon. Orthopedic surgery was consulted and they requested magnetic resonance imaging of both upper extremities. The MRI of the left elbow showed an avulsion fracture at the distal triceps insertion, with 2.6 cm proximal retraction of the triceps tendon. In addition, there was an ulnar collateral ligament was noted to strip off the corresponding tubercle. The MRI of the right elbow demonstrated an acute rupture of the triceps tendon with an avulsion fracture of the olecranon. There was no dislocation noted. Orthopedic surgery subsequently placed bilateral long arm splints on the patient and he was discharged from the emergency department (Figures 1, 2, 3, 4, 5, and 6).

He was subsequently seen by orthopedic surgery as an outpatient and successfully had each triceps tendon sutured to bone bilaterally, with no complications. The patient successfully resumed his career as a police officer with no limitations.

## 2. Discussion

Among tendon injuries, traumatic ruptures of the triceps are rare [1]. There have been less than 80 reported cases in the literature and some have purported that triceps tendon rupture is perhaps the rarest of all tendon ruptures [2]. Simultaneous triceps tendon rupture is even rarer. There have been multiple etiologies of triceps ruptures noted in the literature. Hypocalcemia-induced tetany has been described as one of these etiologies [1]. In addition, metabolic derangements have been noted to be another cause of triceps tendon rupture. The metabolic derangements have been typically chronic in nature and include chronic renal failure, especially in those patients on hemodialysis [3]. The effects of chronic acidosis present in chronic renal failure has been studied and has been shown to be associated with the elastosis of connective tissue [4, 5]. A link to secondary hyperparathyroidism has also been noted [6]. Though there have been only a few cases of tendon rupture secondary to renal failure and secondary hyperparathyroidism reported, only rarely have these authors reported bilateral and simultaneous triceps tendon rupture. The putative mechanism for tendon rupture with renal failure and hyperparathyroidism includes parathyroid hormone-induced breakdown of bone matrix and increases bone resorption, thus weakening the bone-tendon interface [7, 8]. In addition to renal failure, other predisposing factors for this injury have been reported. These include anabolic steroid use and olecranon bursitis [9, 10]. Tendon injuries have also been reported in association with rheumatoid arthritis and systemic lupus erythematosus, though these ruptures tend to occur in sites other than the triceps [11, 12]. 

The majority of triceps tendon ruptures have been noted to occur in healthy tissue without any predisposing factors. The injury typically occurs after a fall on outstretched hand or a direct trauma to the arm. However, the presence of bilateral and simultaneous triceps tendon rupture in a patient without any predisposing factors to our knowledge has not been reported. Other etiologies of triceps tendon rupture in those without specific predisposing factors have included swinging a baseball bat and bench pressing heavy weight. In those injuries secondary to bench presses in the majority of instances, the patients admitted to using anabolic steroids. Furthermore, these cases can be associated with radial head fractures [13]. In our case, the patient had no predisposing factors and had testing performed that ruled out thyroid, autoimmune, calcium, and renal abnormalities.
